# Laponite for biomedical applications: An ophthalmological perspective

**DOI:** 10.1016/j.mtbio.2023.100935

**Published:** 2023-12-28

**Authors:** Maria J. Rodrigo, Maria J. Cardiel, Jose M. Fraile, Jose A. Mayoral, Luis E. Pablo, Elena Garcia-Martin

**Affiliations:** a Department of Ophthalmology, Miguel Servet University Hospital, Zaragoza, Spain; b Aragon Institute for Health Research (IIS Aragon), GIMSO Research Group, University of Zaragoza (Spain), Avda. San Juan Bosco 13, E-50009 Zaragoza, Spain; c Department of Pathology, Lozano Blesa University Hospital, Zaragoza, Spain; d Institute for Chemical Synthesis and Homogeneous Catalysis (ISQCH), Faculty of Sciences, University of Zaragoza–CSIC, C/Pedro Cerbuna 12, 50009 Zaragoza, Spain; e Biotech Vision SLP (spin-off Company), University of Zaragoza, Spain

**Keywords:** Laponite, Clay, Biomedical application, Review, Ophthalmology

## Abstract

Clay minerals have been applied in biomedicine for thousands of years. Laponite is a nanostructured synthetic clay with the capacity to retain and progressively release drugs. In recent years there has been a resurgence of interest in Laponite application in various biomedical areas. This is the first paper to review the potential biomedical applications of Laponite in ophthalmology. The introduction briefly covers the physical, chemical, rheological, and biocompatibility features of different routes of administration. After that, emphasis is placed on 1) drug delivery for antibiotics, anti-inflammatories, growth factors, other proteins, and cancer treatment; 2) bleeding prevention or treatment; and 3) tissue engineering through regenerative medicine using scaffolds in intraocular and extraocular tissue. Although most scientific research is not performed on the eye, both the findings and the new treatments resulting from that research are potentially applicable in ophthalmology since many of the drugs used are the same, the tissue evaluated in vitro or in vivo is also present in the eye, and the pathologies treated also occur in the eye. Finally, future prospects for this emerging field are discussed.

## Graphical abstract

**Figure:**
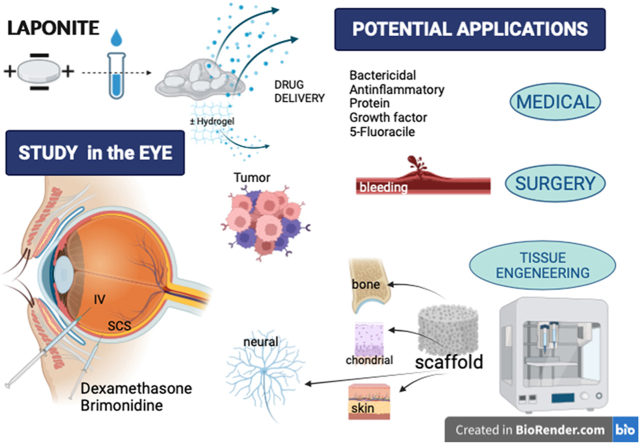

## 1 Introduction

Clay minerals' biocompatibility and nanoscale make them an emerging class of biomaterials suitable for a wide range of biomedical applications. To date (2023), the terms “clays” and “biomedical applications” have been referenced in more than a thousand publications. Clay minerals, however, are not new [1]. Their powerful colloidal properties have been known since 2500 BCE and they have long been used to prevent and treat bleeding [2], skin wounds [3], and gastrointestinal diseases, as well as in cosmetics and personal care products. Several types of mineral clay are currently used in biomedical applications. Natural clays, such as montmorillonite, kaolinite, and halloysite, among others, are abundant in nature and easily obtained, although in general they contain different types of impurities, such as non-clay-mineral particles like quartz and calcite [4]. Synthetic clays like Laponite, meanwhile, are free of impurities and therefore have a more uniform structure and composition. A search for “Laponite” in the PubMed database revealed an increase between 2007 and 2023 in the number of published papers containing this term, demonstrating that Laponite's unique characteristics have made it an attractive biomaterial in recent years. Other emerging materials also have potential biomedical applications. Layered double hydroxides are quite similar to synthetic clays as regards the preparation method. They differ, however, in their greater compositional variability, layered structure, and possible non-covalent interaction with drugs [5]. Their particle size is also usually larger than Laponite's, which has rheological consequences when forming gels. Likewise, their layer charge is positive, in contrast to Laponite's negative charge, leading to different types of electrostatic interaction with drugs and the ionic medium. While hexagonal boron nitride is another promising nanomaterial, preparing it in a form suitable to produce stable colloidal suspensions is challenging [6]. Molybdenum sulfide (MoS_2_) nanoparticles are also emerging as materials with potential for different biomedical applications, including drug delivery [7]. However, this delivery is usually linked to near-infrared laser irradiation which, combined with the black color of MoS_2_ and the doubts about its biocompatibility, makes it difficult to envisage an ophthalmic application for this material.

Laponite® (trademark of the company BYK Additives Ltd) is a synthetic clay developed in the early 1960s [8] as a rheological additive for pigment dispersions [9]. It was prepared by the co-precipitation method using Mg and Li sources (MgSO_4_ and LiF in the first reports [10]), together with a silicon source (sodium silicate) in a basic medium. Improvements to this method allowed preparation to take place in the absence of fluorine, producing different hydrophilicity/hydrophobicity and rheological behavior [11]. Since then, Laponite has not only been used in a variety of industrial applications but has also been subject to extensive research in relation to biomedical applications [[12], [13], [14]]. The translational research performed with Laponite has mainly centered on wound healing; drug delivery systems (small molecules and, more recently, protein delivery) to treat infections, bleeding, or cancer; and tissue engineering for bone scaffolds [15,16]. Very few references to ophthalmological applications for Laponite are found in the scientific literature.

From a physical–chemical point of view, the empirical formula of this synthetic nanosilicate is (Na^+^_0.7_ [(Si_8_Mg_5·5_Li_0.3_)O_20_(OH)_4_]^−^_0.7_ [17]. As a clay mineral, its basic building blocks consist of alternating tetrahedral SiO_4_ and octahedral AlO_6_ sheets in a 2:1 ratio [4,18]. This means it comprises two tetrahedral silica sheets positioned on either side of an octahedral sheet bound through shared oxygens [19], thus forming a layered structure. Laponite has a dual-charged surface. The faces (upper and lower surfaces) are negatively charged due to the charge imbalance caused by magnesium substitution by lithium in the octahedral layer, while particle edges can be positively charged by protonation of the terminal hydroxyl (OH) groups of the tetrahedral silicate layers. Several layers may be stacked one on top of the other, mainly by electrostatic force, but also hydrogen bonding and Van der Waals force, and joined in clay crystallites with interlayer cations [20]. Layered silicate clays offer a high surface area (more than 700 m^2^/g) and allow drug, polymer, protein, or extracellular vesicle interaction and retention to occur, thus forming multifunctional drug delivery systems for better pharmaceutical performance.

In dry form, Laponite has a two-dimensional (2D) disc-shaped geometry (diameter 20–50 nm and thickness approximately 1–2 nm). However, in water this becomes three-dimensional (3D) colloidal particles of colorless gel (the ‘house of cards’ structure or ‘T configuration’) [9]. Microenvironmental conditions such as pH or salt concentration have a significant impact on gelation time. The charge on the edges of the Laponite is pH-dependent because of protonation of exposed hydroxyl groups. Gelation time is found to increase significantly with decreasing salt and Laponite concentration. Conversely, an increase in ionic strength leads to the formation of aggregates. Gelation happens abruptly and precipitates when salt concentration exceeds 11 mM; however, with Laponite concentrations above 10 g/L salt concentration does not impact gelation time. The house-of-cards structure is obtained at lower salt concentrations or at pH values below 11 and is generated by preferential interaction between the negative charge on the basal plane and the positive charge on the edge of the particles. At higher salt concentrations band-type aggregates or stacked configurations can be generated by face-to-face interactions with cations, which can later transition into the house-of-cards structure. Other environmental changes, such as local humidity, can cause the clay to absorb or lose water, resulting in variable swelling [20]. The permeability to water and the diffusion of small molecules depend on the orientation of the clay particles within the gel. Well-oriented particles can be used as a barrier to gases and liquids, while randomly or haphazardly oriented particles can increase permeability [21]. Swelling can be prevented by the formation of interactions between polymers and Laponite, controlling in this way the slow release [[22], [23], [24]].

Nanosilicates are optically transparent in aqueous media. Laponite can be functionalized with fluorophores, luminescence, and paramagnetic particles [25,26]. These characteristics can be beneficial, especially in ophthalmic applications, since they enable imaging of subsurface cellular behavior, design of complex printed tissues [27], and facilitate optical coherence tomography (OCT) and magnetic resonance imaging (MRI) for monitoring, diagnosis, and treatment of pathologies [28,29].

Laponite is a rheology modifier commonly used to adjust the overall viscosity of the drug formulation and to control non-Newtonian behaviors such as shear thinning or shear thickening [30,31]. This thixotropic property of Laponite [32] facilitates injectability using small-gauge needles and improves shear-thinning behavior, which is highly desirable for injectable hydrogel devices in which encapsulated drugs such as proteins or antibodies and/or cell activity must be preserved under the high shear stress exerted by injection [33].

**Fig. 1:**
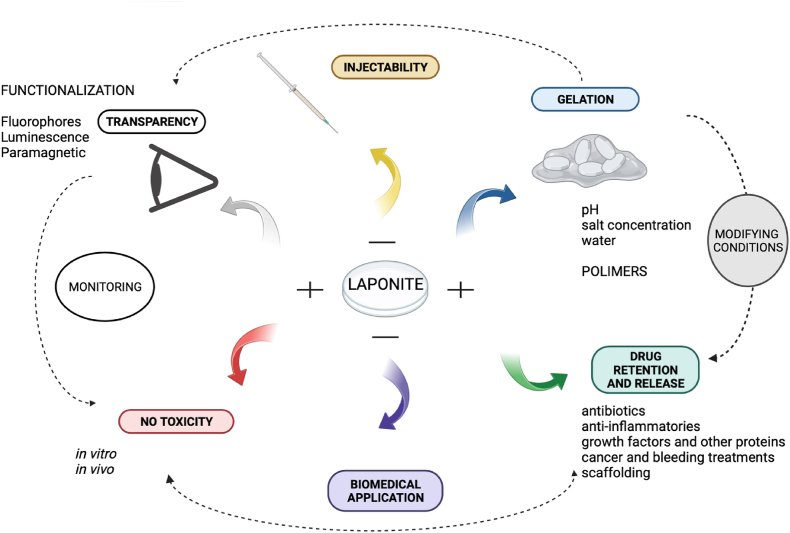
Laponite properties for ophthalmological applications. Created in BioRender.com.

Biocompatibility is key for medical translation. Based on experimental and modeled data, the U.S. Environmental Protection Agency has verified that Laponite is a safe chemical of low concern. A range of studies have demonstrated the high biocompatibility of Laponite, establishing its widespread biomedical application. Laponite does not present systemic toxicity after oral, intramuscular, or ocular administration [[34], [35], [36], [37], [38], [39], [40]] at low concentrations (0.1–7% w/v) or with an inhibitory concentration (IC50) of 4 mg/mL [41]. In addition, after intravenous administration a nanocomposite based on Laponite exhibited no hemolytic activity in vitro and no histopathological alterations in the brain, heart, liver, or kidney tissues of mice in vivo [42]. However, prolonged oral medication is not recommended due to the risk of kidney stone formation and the elimination of enzymes and other nutritive elements. Furthermore, high concentrations of nanosilicates can reduce cell proliferation in vitro [43]. Several studies claim IC50 values of Laponite vary considerably, ranging from 0.05 to 50 mg/mL [44]. Laponite particles have been shown to naturally degrade in ∼30 days on average; a hydrogel with a residence time of >30 days prevents complete Laponite particles escaping the hydrogel, thereby preventing adverse cytotoxicity [45]. Laponite, especially LaponiteⓇ XLG, is considered suitable for biomedical application due to its low heavy metal content. This hydrous nanosilicate contains elements such as magnesium, zinc, lithium, and iron that are also found in the body and in brain metabolism, and its nontoxic degradation products [Na^+^, Mg^2+^, Si(OH)_4_, Li^+^] are easily absorbed by the body [42,46]. Fig. 1.

This review examines the scientific evidence currently available on Laponite in ophthalmology. It also focuses on potential applications in the eye that can benefit from the promising opportunities offered by innovative Laponite-based biomaterials. It looks at the biomedical applications of Laponite in 1) drug delivery of antibiotics, anti-inflammatories, growth factors, other proteins, and cancer treatments; 2) bleeding prevention or treatment; and 3) tissue engineering through regenerative medicine using scaffolds.

## 2 Methodology

A systematic search was conducted using the Preferred Reporting Items for Systematic Reviews and Meta-Analyses (PRISMA) guidelines for structured reviews. The literature search was carried out on platforms such as Web of ScienceⓇ, the Wiley database, https://www.sciencedirect.com/topics/biochemistry-genetics-and-molecular-biology/scopusⓇ, Google ScholarⓇ, Pubmed, PubChem, MendeleyⓇ and the science.gov databases as at August 2023. For the literature search, different combinations of keywords, such as “clay minerals”, “Laponite”, “biomedical applications”, “review”, and “ophthalmology” were used. The identification, screening, eligibility, and inclusion of scientific evidence are shown in Table 1.

**Table 1:** Search sequence for the selection of studies considered for review. * The complete list of studies is provided in the supplementary material.

**Database**	“Laponite” and “review” (since 1992)	“Laponite” and “biomedical applications” (since 2009)	“Laponite” and “biomedical applications” and “review” (since 2017)	“Laponite” and “ophthalmology” (since 2018)	Screening
Google Scholar	>3000	>1000	>1000	114	**Identification**
Web of Science	155	81	16	1	
PubMed	22	45	12	5	
Google Scholar	173	126	126	7	**Eligibility**
Web of Science	26	52	10	1	
PubMed	17	39	12	4	
After eliminating repetitions	188	179	131	11	**TOTAL** 242 *

The studies identified, from general to specific, were those containing the following: the general term [“clay minerals”], which found 48 521 matches in Web of Science, 14 400 in Google Scholar, and nearly 3000 in both Pubmed and PubChem (since 1915); the specific term [“Laponite”], which found more than 2700 matches in Web of Science, more than 2200 in Google Scholar, and around 700 results in Pubmed (since 1969); and [“Laponite NM”], which returned a total of 187 results in Pubmed and 179 consolidated references in PubChem (since 2007). As can be seen in Table 1, using Boolean operators to combine the selected terms [“Laponite” and “review”], [“Laponite” and “biomedical applications”], [“Laponite” and “biomedical applications” and “review”], and [“Laponite” and “ophthalmology”] to focus more specifically on the ophthalmic application of Laponite produced fewer matches, which were found in more recent scientific papers.

For the screening step, all the titles obtained from all the database searches using the Boolean operators and keywords [“Laponite” and “review”], [“Laponite” and “biomedical applications”], [“Laponite” and “biomedical applications” and “review”] and [“Laponite” and “ophthalmology”] were read. For those references that met the exclusion and inclusion criteria, respectively, the abstract was read in the eligibility step. The exclusion criteria were 1) communications, abstracts, or studies with little scientific evidence (i.e. not included in the Journal Citation Reports (JCRⓇ) database) on mineral clays and not mentioning Laponite, 2) references focused solely on chemical properties, or 3) industrial applications, and 4) references in non-English languages. The inclusion criteria were studies or reviews focused on 1) Laponite clay in general terms, 2) clay minerals, including Laponite, for biomedical applications, and 3) the use of Laponite in the eye. If the paper was considered of special interest as regards ophthalmic translation or application, or as regards the perspective offered, the complete paper was read in detail and incorporated in this review.

For the inclusion step, the studies selected were classified based on whether they were 1) conducted in the eye or 2) not conducted in the eye but the drugs are also used in the eye, the tissue evaluated in vitro or in vivo is also present in the eye, the pathologies treated also occur in the eye, and/or the new treatments are potentially applicable in the eye. Accordingly, topics relating to drug delivery, bleeding and tissue engineering using regenerative medicine and scaffolds were discussed.

## 3 Results

**Fig. 2:**
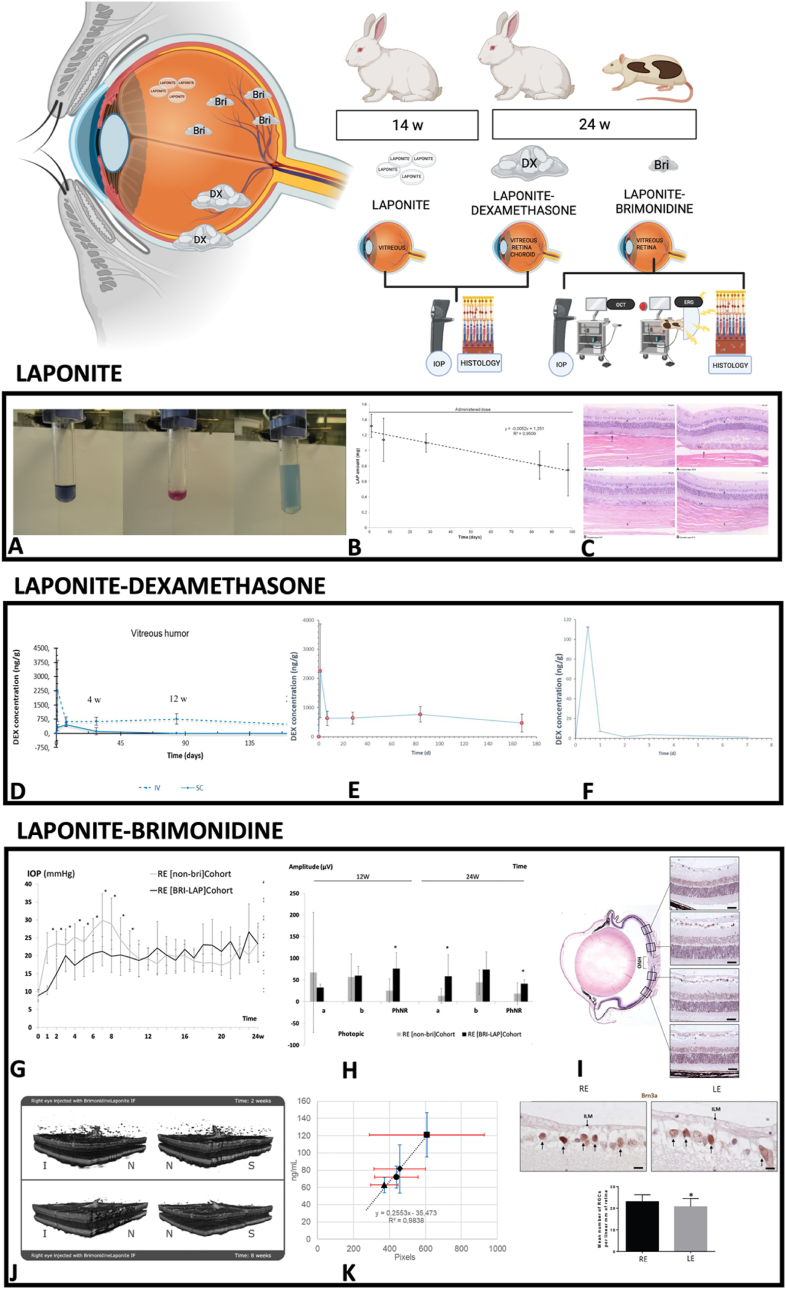
Studies of Laponite conducted in the eye. Abbreviations: IOP: intraocular pressure; OCT: optical coherence tomography; ERG: electroretinography; w: weeks. Created in BioRender.com. **A**: Complexometric determination of Mg2+ in rabbit vitreous humor. Left: Eye not injected with Laponite dispersion: the blue color after addition of EBT indicates the absence of magnesium. Middle: Eye injected with Laponite dispersion: the pink color after addition of EBT indicates the presence of magnesium. Right: Color change in vitreous sample after titration with the required volume of EDTA. **B**: Laponite levels over time in the vitreous humor of intravitreous administered eyes. **C**: Representative photomicrographs showing histological sections of the retina from treated (left panel) and control eyes (right panel) at 14 weeks after injection (HE; magnification Å∼ 650). Top: Suprachoroidal administration. Bottom: Intravitreal injection. All retinal layers are preserved and no differences between treated and control eyes were observed in the 20-step sections assessed. The ganglion cell layer is facing the upper side of the photograph. In suprachoroidally administered eyes, virtual spaces can be seen at the junction line between the choroid and sclera (arrows). R: retina; Ch: choroid; and S: sclera. A, B and C: Data from Ref. [40] (CC BY 4.0 license). **D**: Dexamethasone concentrations in the vitreous humor after intravitreal and suprachoroidal administration of Laponite–Dexamethasone (1:10 w w−1) suspension (10 mg mL−1). **E**: Dexamethasone concentrations in the vitreous humor after intravitreal administration of Laponite–Dexamethasone suspension. **F**: Dexamethasone concentrations in the vitreous humor after intravitreal administration of Dexamethasone solution. D, E and F: Data from Ref. [48] (CC BY 4.0 license). **G**: Intraocular pressure curves. Eye comparison between the [non-BRI] cohort (rats with ocular hypertension) and the [Bri-Lap] cohort (rats with ocular hypertension and treated with an intravitreal injection of Laponite–Brimonidine formulation). **H**: PhNR amplitude (a and PhNR waves) was statistically significantly higher in eyes treated with the Bri-Lap formulation in comparison with hypertensive and untreated eyes in the [non-Bri] cohort. Abbreviations: RE: right eye; a wave: signal from photoreceptors; b wave: signal from intermediate cells; PhNR wave: signal from retinal ganglion cells. w: week; μV: microvolts; *p < 0.05: statistical differences. **I**: Retinal ganglion cell analysis in glaucomatous eyes. Top: Retinal ganglion cells were counted in radial sections of the eye along 2 mm of a linear region of the retina, corresponding to four areas, two on each side of the optic nerve head. Middle: Two representative images of the ganglion cell layer marked with anti-Brn3a corresponding to a treated (RE) and non-treated eye (LE) of the same animal. Arrows mark the positive nuclei. Bottom: The mean number of retinal ganglion cells per linear mm of retina was significantly higher in hypertensive eyes injected with Laponite–Brimonidine formulation than in the untreated eyes (RE 23.00 ± 0.39 vs. LE 20.66 ± 0.98, p = 0.040). Abbreviations: RE: right eye; LE: left eye; ILM: internal limiting membrane. Scale bars: top: 22.72 μm; middle: 5.8 μm. G, H and I: Data from Ref. [49] (CC BY 4.0 license). **J**: 3D reconstruction of the changes in the aggregates at 2 weeks and 8 weeks of follow-up. The reconstruction is shown from two different perspectives at each point in time. Abbreviations: N: nasal; I: inferior; S: superior; IF: intravitreal formulation. **K**: Positive linear correlation between drug levels and total aggregate area. OCT data in red; brimonidine data in blue; ■: 1 week;: 4 weeks; ●: 8 weeks; ▲: 24 weeks. J and K: Data from Ref. [28] (CC BY 4.0 license).

In total, 173 publications were included in this review, around 80 % of which were published in the last 10 years (between 2013 and 2023) (Table 2 and Supplementary Table 1). Regarding studies conducted in or for the eye, we found 11 publications focusing on “Laponite” and “ophthalmology”. However, only 4 of the 11 were conducted on in vivo animal eyes (on healthy rabbit eyes and on rats with induced glaucoma, and by two different routes of administration: suprachoroidal, performed surgically; and intravitreal, by minimally invasive injection). After suprachoroidal and intravitreal administration in rabbit eyes, Laponite exhibited biocompatibility since there were no significant differences in intraocular pressure, no relevant ocular complications were found after either route of administration, and no pathological changes were observed in histology. In addition, slow degradation of Laponite was observed over 14 weeks. Laponite presence in the vitreous was indirectly confirmed by complexometric titration taking advantage of Laponite's high magnesium ion content [40]. Thereafter, controlled in vitro delivery of dexamethasone was evaluated in solutions used as models for the vitreous humor. This study highlighted the simplicity of the preparation method, in which physisorption was modulated by changing the solvent in the adsorption process [47]. The same study group also exhibited good tolerance and sustained-release delivery of two drugs (dexamethasone and brimonidine) commonly used in ophthalmology. A dexamethasone–Laponite formulation was obtained from the interaction between the non-ionic drug and Laponite, mostly by hydrogen bonding involving hydroxyl and carbonyl groups and, after suprachoroidal and intravitreal administration in healthy rabbit eyes, was well tolerated; dexamethasone levels in the choroid–retina unit and vitreous were detected up to 24 weeks. It concluded that Laponite increased the intraocular retention time of dexamethasone when compared with conventional solutions [48]. Intravitreal injection is an administration route commonly used in ophthalmology to maintain therapeutic drug levels near the neuroretina when treating pathological conditions. In glaucomatous rat eyes, a brimonidine–Laponite formulation injected into the vitreous induced an ocular hypotensive and neuroprotective effect, corroborated over 24 weeks by electroretinography, OCT, and higher retinal ganglion cell counts using immunohistochemistry; the authors even observed delayed bilateral neuroprotection [49]. Furthermore, the brimonidine–Laponite formulation was monitored noninvasively using vitreoretinal interface imaging captured with OCT [28]. The formulation was identified as vitreous hyperreflective aggregates which correlated with brimonidine levels measured in the eye. The other publications found were studies or reviews citing the aforementioned [15,[50], [51], [52], [53], [54], [55], [56]]. (Fig. 2).

**Table 2:** Summary of studies involving Laponite conducted in or for the eye in the last 10 years (2013–2023).

PUBLICATION	STUDY	MOLECULE	ROUTE	OUTCOMES
Article	*In vitro & In vivo*	Brimonidine	Intravitreal administration	Brimonidine–Laponite treatment for glaucoma can be monitored non-invasively using vitreoretinal interface imaging captured with optical coherence tomography over 24 weeks of follow-up and correlated with brimonidine levels measured in rat eyes [28].
Article	*In vivo*	–	Intravitreal & suprachoroidal administration	Safety and biocompatibility of Laponite clay in rabbit eyes [40].
Article	*In vitro*	Dexamethasone	Vitreous humor models	Laponite clay can retain dexamethasone by simple physisorption and deliver it in a controlled manner in solutions used as models for the vitreous humor. It is transparent in the gel state, and the preparation method is simple [47].
Article	*In vitro & In vivo*	Dexamethasone	Intravitreal & suprachoroidal administration	Sustained-release delivery of dexamethasone using Laponite as a carrier after intravitreal and suprachoroidal administration in rabbit eyes over 14 weeks [48].
Article	*In vitro & In vivo*	Brimonidine	Intravitreal administration	A brimonidine–Laponite intravitreal formulation has an ocular hypotensive and neuroprotective effect throughout 6 months of follow-up in glaucomatous rats [49].
Review	*In vivo*	–	Suprachoroidal administration	Delivery of existing and novel therapeutic agents, such as Laponite, into the potential space between the sclera and choroid and a promising drug delivery route to the posterior segment of the eye [50].
Editorial	*In vitro & In vivo*	Brimonidine	Intravitreal administration	The most recent cutting-edge research in ophthalmic drug delivery, highlighting a glaucoma treatment combining a hypotensive and neuroprotective intravitreal formulation of brimonidine–Laponite that could be monitored non-invasively using optical coherence tomography [51].
Review	*In vitro & In vivo*	–	–	Summarizes recent findings and patents on various nanotechnology products, such as Laponite, in ocular drug delivery [52].
Review	*In vitro & In vivo*	–	–	Natural and synthetic clays for drug delivery and tissue engineering applications from in vitro/in vivo studies [53].
Review	*In vitro & In vivo*	–	–	New three-dimensional delivery strategies, including Laponite, for growth factors show promise compared to conventional methods [54].
Review	*In vitro & In vivo*	–	–	Applications on biological cationic mineral clay systems [55].
Review	*In vitro & In vivo*	–	–	Hydrogels including Laponite for drug delivery and biomedical devices among several applications [56].

**Fig. 3:**
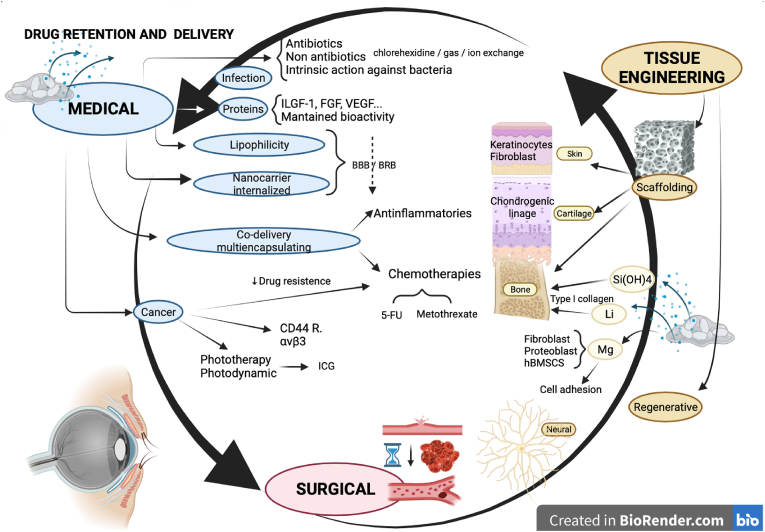
Studies of Laponite not conducted in the eye, potentially applicable in ophthalmology. Abbreviations: ILGF-1: insulin-like growth factor-1; FGF: fibroblast growth factor; VEGF: vascular endothelial growth factor; BBB: blood–brain barrier; BRB: blood–retina barrier; 5-FU: 5-fluorouracil; ICG: indocyanine green; Si(OH)4: Orthosilicic acid; Li: Lithium ions; Mg: Magnesium ions. Created in BioRender.com.

The following scientific studies were not conducted in the eye, but they could be potentially applicable in ophthalmology since the same drugs are used, the tissue evaluated in vitro or in vivo is also present in the eye, the pathologies treated also occur in the eye, and/or the new treatments are potentially applicable in the eye. To examine the broad spectrum of biomedical applications of Laponite in the eye, and as ophthalmology is a medical and surgical specialty, the following studies related to drug delivery (for medical issues), bleeding (for surgical issues), and tissue engineering with regenerative medicine and scaffolds (for minimally invasive repair of the eye in prospective applications) are referenced (Fig. 3 and Supplementary Table 1).

1) **Drug retention and delivery** [57,58]. Modern drug delivery technology is only 60 years old. The first generation (1950–1980) established the basis for controlled release and successfully managed delivery systems' physical–chemical properties. The second generation developed smart delivery systems but struggled with biological barriers. Today's third generation (2010–2040) comprises modulated delivery systems designed to overcome both the physical–chemical and biological barriers [59] in the eye with the objective of administering therapeutic drug levels with minimum intervention.

Drug molecules are classified as either small or large (molecular weights 12–35 kDa) or biologics [60]. The main mechanism of Laponite drug uptake is intercalation. However, some fractions can be adsorbed on the surface of the particle. The combination of high surface area and charge also results in sustained release of the loaded therapeutics [61]. Moreover, as Laponite is highly hydrophilic it can easily interact with a range of polymeric hydrogels and cryogels, inhibiting burst lease [60,62]. Recently, Laponite-loaded polymeric hydrogels received approval from the U.S. Food and Drug Administration, thereby establishing their clinical potential [63]. Laponite nanodiscs exhibit pH- and salinity-dependent drug loading and release behavior, in which higher swelling and an acidic environment lead to faster release [64]. Laponite particles naturally dissociate into their constituent ions (Li^+^, Mg^2+^, and Si(OH)_4_) in environments where the local pH is less than that of the isoelectric point of Laponite (pH ∼10), degrading the nanosilicate particles in about 20–50 days [65]. It is therefore useful at the low pH values that are typically observed in inflamed, ischemic, and neoplastic tissue. However, it may be not favorable where physiological pH is required for the release of the molecule. In acidic conditions, the surface of Laponite is more positive, forming very strong bonds with negative substances. In neutral or basic pH conditions, Laponite maintains a negative surface by adsorptive binding. Neutral molecules may still interact with Laponite particles if their charge is anisotropically distributed [66]. This wide variety of possible bonds means Laponite has been used in a variety of studies on the delivery of both small and large molecules.

In this regard, the small molecules intercalated with Laponite for infection and inflammation prevention or treatment were antibacterial agents such as tetracycline [67], amoxicillin [68], ofloxacin, ciprofloxacin [69], vancomycin, and mafenide [70], which all exhibited extended release. Other alternatives to infection and inflammation prevention or treatment were chlorhexidine [71], gas [72], and ion exchange [73,74]. Interestingly, Laponite seems to have a specific action against Gram-negative bacteria. Anionic Laponite nanoparticles were able to effectively aggregate Gram-negative bacteria through lipopolysaccharide binding, which decreased bacterial cells. This suggested Laponite to be beneficial in confining bacterial infection and inflammation [75]. In addition, anti-inflammatory agents such as theophylline and vitamin B12 were co-delivered [76], and dexamethasone exhibited sustained release in vitreous gel in rabbit eyes [48].

Eye and brain disorder therapy nevertheless remains challenging, partly because of the existence of the blood–retina barrier and the blood–brain barrier (BRB and BBB) [77]. In this regard, drug lipophilicity is an important parameter that determines a drug's capacity to penetrate those barriers. Laponite enhances the solubility of non-water-soluble drugs such as the antifungal itraconazole [78], the neuroprotector brimonidine (intravitreal) [28], ITH12657 (oral) [79], and donepezil (intravenous), administration of which improves delivery of either the therapeutic agent [42] or magnetic nanoparticles [80].

Regarding large molecules, the existence of hydrophilic and hydrophobic regions on the clay's surface facilitates protein molecules' interaction with the clay [81,82]. Proteins and other macromolecules can form relatively large complexes and interact with Laponite particles via face-only or edge-only interactions. Laponite–protein complex size increased with Laponite concentration due to the increase in surface area available for adsorption. Positively charged proteins (such as ribonuclease A or lysozyme) form larger complexes with Laponite and are released much more slowly than negatively charged proteins (such as bovine serum albumin). Laponite mitigated the burst release for proteins and extracellular vesicles [83,84] and allowed tunable release times. A Laponite–insulin-like growth factor-1 (ILGF-1) mimetic protein hydrogel [85] exhibited release up to 4 weeks in a rat model. As protein secondary and tertiary structure is very important for bioactivity, protein structure must be preserved following interaction and release from Laponite [60]. The maintenance of protein bioactivity was demonstrated in proteins such as albumin and lysozyme, and in growth factors such as transforming growth factor-β3 (TGF-β3), human mesenchymal stem cell-derived growth factors, fibroblast growth factor 2 (FGF2), and vascular endothelial growth factor (VEGF) [86], as well as in heparin-fibroblast growth factor 4 (FGF4) [87], human bone morphogenetic protein 2 (rhBMP2) [88], and IGF1 [85] in vitro and in vivo [31,87,[89], [90], [91], [92]] —the latter focused on wounds or on spine, bone, or tendon injuries— and achieved desired physiological outcomes. However, further characterization and understanding of the Laponite–protein complex structure when used with inflammatory cytokines such as granulocyte macrophage colony-stimulating factor (GM-CSF), FMS-like tyrosine kinase-3 ligand (FlT3L), Interleukin (IL)-15, IL-2, or chemokine ligand 20 (CCL20) is necessary. At the same time, delivery of macromolecules other than proteins such as immunoglobulins, which are highly important in ophthalmology (as anti-VEGFs), presents further challenges and should be explored.

A separate point to be considered, given its significance and the evidence available, is anti-cancer therapy [93]. Nanoclays are emerging as systems offering extraordinary potential in cancer theranostics, not only as vectors for the delivery of different anti-cancer agents with intrinsic anti-tumor activity, but also in diagnosis [94,95]. Laponite can be physically triggered by temperature and magnetic/electric or light fields, which is useful in optical therapies, including photothermal therapy (PTT) and photodynamic therapy (PDT). Meanwhile, loading chemotherapeutics such as doxorubicin into the interlayer space of Laponite particle gels demonstrated the utility of this nanoclay for delivery applications [[96], [97], [98]], while doxorubicin exhibited higher release in the acidic environment of tumors [99]. Furthermore, Laponite served as a nanocarrier across the cellular membrane in a doxorubicin-loaded nanocomposite hydrogel versus a bolus drug dose [100]. In vivo, Laponite particles are thought to be internalized by clathrin-mediated endocytosis and subsequently degraded within the low pH environment of endosomes [101]. This internalization was also suggested after OCT visualization [28]. In addition, an antimelanoma Laponite gel formulation containing simvastatin [102], a physical crosslinking of magnetic Laponite nanoparticles with 5-fluorouracil [103]— and methotrexate, which is also used for inflammatory pathologies affecting the eye [104], exhibited antitumoral activity. Finally, benzoyl peroxide generated and sustainedly released oxygen, which chemically modified the tumor microenvironment and reversed the effects of hypoxia. Consequently, the proliferation of malignant cells decreased while the viability of healthy fibroblasts increased [105]. There are also new strategies to enhance the efficacy of chemotherapy. Co-loading or simultaneously multiencapsulating different drugs (chemotherapies) in Laponite improved their efficacy versus their effectiveness individually [97], and co-delivery with sequential release overcame tumor drug resistance [106]. In this regard, a promising cancer treatment target is CD44, a transmembrane glycoprotein overexpressed in several solid tumors such as melanoma, non-Hodgkin's lymphomas, gliomas, or meningiomas, all of them also present in the eye. Adding CD44-targeting receptors to other chemotherapies significantly enhanced antitumor activity [107]. Another example, as mentioned before, is a photothermal and photodynamic Laponite-based therapeutic agent for the treatment of cancer cells overexpressing integrin αvβ3, which involved applying a coating of polydopamine (PDA) to indocyanine green (ICG)-loaded Laponite and then conjugating polyethylene glycol-RGD (PEG-RGD) on the surface. Indocyanine green is a photothermal dye widely used in ophthalmology for choroidal pathologies [108]. ICG encapsulation efficiency was 94.1 % and the photostability of the ICG protected with Laponite and PDA was dramatically improved. This combined drug enhanced cellular uptake by cancer cells overexpressing integrin αvβ3 and caused cell death under in vitro near-infrared laser irradiation by generating reactive oxygen species [109]. Furthermore, Laponite exhibited luminescent properties, producing bright red and bright green emissions [26], or was functionalized by fluorophores [25,110], thereby suggesting its utility for imaging diagnosis.

In addition to serving as a delivery system for the molecules mentioned above, Laponite has long been known for its hemostatic ability. Studies potentially applicable to the prevention or treatment of bleeding in surgical settings are briefly referenced below.

**2) Bleeding:** Hematotoxicity is one of the factors limiting in vivo use of biomaterials. Hemolysis and coagulation induced by clay particles offer a possible use of these nanocomposite systems in vivo [111,112]. A shear-thinning nanocomposite hydrogel composed of synthetic silicate nanoplatelets and gelatin promoted coagulation. The combination of injectability, rapid mechanical recovery, and physiological stability resulted in a promising hemostat with which to treat incompressible wounds. It would therefore be beneficial in the case of retrobulbar bleeding, in which compression would be forbidden in an open eye so as to avoid iatrogenic optic neuropathy. The tendency of these clays to elicit a procoagulant response depends on the structural and surface properties of the clay. Magnesium aluminum hydroxide layers might limit hemorrhage via adhesion to tissues and red blood cells. As calcium and magnesium ions are required for certain enzymatic reactions in the coagulation cascade, their delivery by materials might therefore influence coagulation kinetics. Factor XII activation correlates with negative surface charge density. Laponite's ability to readily absorb water and swell upon hydration may thicken the blood and restrict blood flow. Furthermore, addition of Laponite to hydrogels used as an injectable hemostat provided enhanced physiological stability and accelerated clotting time by increasing platelet binding and therefore reducing hemostatic clot formation time from 7 min (normal physiological process) to less than 3 min. Sustained release of entrapped therapeutics (VEGF) also promoted enhanced wound healing [111,113]. The incorporation of Laponite into a gelatin hydrogel improved antithrombogenicity and hemocompatibility, and incorporation into a dextran-based hydrogel did not significantly alter hemolysis. Wang et al. found that Laponite particles presented <5 % hemolysis, which could be improved by sintering Laponite particles at high heat. Meanwhile, a mixed suspension containing Laponite and gelatin and incorporated into the polymerization of the acrylamide network resisted nonspecific protein adsorption, improved the degree of hemolysis, and eventually prolonged clotting time [39,69,114]. Thus, hemolysis and coagulation should not be a major concern with Laponite–hydrogel composites, as demonstrated by their good blood compatibility [39,69,114].

Finally, two novel therapies with potential application in several areas of ophthalmology are presented.

3) **Tissue engineering: regenerative medicine and scaffolds**. Laponite has been used for numerous tissue engineering applications. Tissue engineering is a relatively new field where science and engineering work together to reach new frontiers in regenerative medicine. It employs scaffolds, biodegradable structures seeded with human cells and growth factors to develop new tissue while the scaffolds themselves degrade [115]. Meeting the growing demand for personalized implants and tissue scaffolds requires the advanced biomaterials and processing strategies that the fabrication of 3D structures entails. Bioprinting is a revolutionary innovation that can generate 3D scaffolds —even with time-dependent transformation of the printed construct (4D)— and achieve expanding patterns [116], mimicking the complexity of the extracellular matrix and providing excellent functional and biological cues for faster tissue regeneration. Laponite offers a promising platform for bioprinting the cells, resulting in cell-laden constructs designed to assist tissue repair and recover functionality [[117], [118], [119], [120], [121], [122]]. In this regard, the shape of the filler has a relevant impact on the mechanical properties and printability of the scaffolds [123,124]. Laponite gels with different morphologies (droplets, rings, strings, and clay microcapsules) were able to flow through syringe needles and re-establish the gel network due to the Laponite's self-assembling property. Printed scaffolds demonstrated excellent shape fidelity up to 2 cm in height and with changed orientations of between 20° and 90° up to 3 weeks, after which their mechanical properties drastically decreased. Mechanical testing revealed that nonporous solid scaffolds had a higher compressive strain than porous ones. However, current research still lacks answers regarding the mechanisms by which nanofiller shape and morphology affect mechanical and rheological properties. Most studies suggest the formation of a house-of-cards structure; however, this effect does not generally arise at the low concentrations usually considered. Our group demonstrated that, when injected, Laponite intravitreous formed a unique clog when using dexamethasone in healthy rabbits [48] but formed multiples and microaggregates when using brimonidine in glaucomatous rats [28], confirming what in vitro studies of Laponite concentrations have previously shown [15,125]. This fact makes it evident that more research into soft-tissue engineering applications is needed [126,127].

Furthermore, appropriate bioink viscosity is critical to cell printing. Cell-laden Laponite-based nanocomposite bioinks demonstrated superior printing properties that enabled the creation of complex forms and the spreading of various encapsulated cells [[128], [129], [130]]. Cell-laden constructs preserved their morphological properties and exhibited good cell viability (70–75 %) for up to 3 weeks [129], although this also decreased when Laponite concentration rose to 1 % [131].

Recent literature in the field of 3D scaffold bioprinting confirms the enormous potential that use of Laponite has for skin, cartilage, and bone repair/regeneration [132]. An advantage of Laponite is that it creates regenerative microenvironments [133]. In this regard, experimental studies using next-generation sequencing technology demonstrated that nanoclays influence genetics [134]. Laponite exhibited cell viability in relation to https://www.sciencedirect.com/topics/engineering/keratinocyte [37] and fibroblast cells [135], stabilized the intrinsic triple-helical conformation of collagen [136], bridged the tissue gaps, and led differentiation towards the chondrogenic lineage when cultured in a chondrogenic-inducing medium [91,137]. However, Laponite dissolution in an aqueous environment also degraded in nontoxic products, such as Si(OH)_4_, Li^+^, and Mg^2+^, which enhanced osteogenic cell function and promoted osteogenesis by influencing nucleation and deposition of inorganic calcium and phosphate ions in an extracellular matrix. Orthosilicic acid stimulates osteoblast differentiation and type 1 collagen synthesis [138]. Lithium ions are known to promote type I collagen and to initiate canonical Wnt-reactive osteogenic genes via glycogen synthase kinase-3 beta (GSK3β) inhibition [139]. It has been shown to impart osteogenic and angiogenic potential [130]. Furthermore, Laponite could enhance bovine serum albumin and VEGF release kinetics. Magnesium ions are engaged in initiating osteogenesis-governing pathways [[140], [141], [142]] and have been shown to promote cell adhesion to biomaterial surfaces [143] by interacting with the adhesion protein of the integrin family, the primary perpetrators of cell adhesion [[144], [145], [146], [147]].

Nanosized Laponite particles can also themselves directly adhere to the cell surface [148,149] and internalize into the cells [41,150], inducing osteogenic differentiation of mesenchymal stem cells without the use of differentiating media [[144], [145], [146],[151], [152], [153], [154]]. Nevertheless, the mechanisms involved in clay-induced osteogenic differentiation are still poorly understood [155]. Schmidt et al. demonstrated increased cell adhesion and flat and well-spread cell morphology after increasing the Laponite content in a nanocomposite film. Laponite inclusion in PEG hydrogels at 40–70 % (wt%) improved cell adhesion and proliferation and the spreading of mouse preosteoblasts [156,157], mouse fibroblasts [158], and human bone marrow stromal cells (hBMSCs) [38] in a clay-concentration-reliant manner.

Furthermore, 3D-printable zwitterionic Laponite hydrogel demonstrated neural cell viability by growing cells with extended neurites [159].

## 4 Discussion

A biomaterial is a material intended to interface with biological systems to evaluate, treat, augment, or replace any tissue, organ, or function of the body and, in the case of the eye, to compensate for vision loss which may or may not be related to age. Ophthalmic biomaterials try to emulate natural materials, and important requirements must be met [[160], [161], [162]]. Compatibility remains a fundamental issue, as does the ability to deliver oxygen to tissue. A refractive index near that of water is also required, which means most materials to be placed in the eye must be transparent, a prerequisite unique to ophthalmic biomaterials. In addition, a combination of surface and mechanical properties that remain stable throughout the application period must also be produced. Lubrication and friction, tissue protection during surgery, tissue integration, and healing modulation are also widely considered to be important. Numerous studies have shown various biomaterials to be highly beneficial in treating ophthalmic conditions. Biomaterials, tissue engineering, and regenerative medicine are therefore becoming increasingly important to advancing ophthalmology and optometry. This review shows the benefits and the potential biomedical applicability of Laponite as an ophthalmic biomaterial. Apart from being biocompatible, easily injectable, and optically transparent, Laponite increases therapeutic delivery and uptake of several drugs used in ophthalmology, with the added advantage of encouraging intrinsic antimicrobial activity by modifying the ionic or oxygen microenvironment. In this regard, since the use of fewer antibiotics is currently supported and encouraged to avoid resistance, Laponite could also have a role in ophthalmology where eye drops based on iodine, ozone, or chlorhexidine have demonstrated their bactericidal efficacy [163]. Laponite achieves sustained release of molecules and even of extracellular vesicles [164] and when combined with other hydrogel polymers can diminish the initial burst. Sustained release has been demonstrated in small antibiotic and anti-inflammatory molecules widely used in ophthalmology, such as tetracyclines, quinolones, or corticosteroids. These drugs are widely used to treat inflammatory processes affecting the palpebral, such as blepharitis or prophylactics for cataract surgery, among others. Two drugs commonly used in medical and surgical glaucoma therapy (brimonidine and 5-fluorouracil) exhibited sustained release and efficacy. Moreover, the sustained release of macromolecules such as growth factors (e.g., mimetic protein ILGF-1) and other proteins (e.g., VEGF) while maintaining functionality has been demonstrated. ILGF-1 could be useful likely as insulin eye drops, which have shown great effectiveness in closure of corneal ulcers [165], with the added advantages of retaining water to prevent desiccation, reducing the number of applications, and maintaining optical transparency. However, we have not been able to find any reference to the combination of Laponite with anti-VEGF antibodies, widely and repeatedly used as a therapeutic target in ocular pathologies of the posterior pole causing blindness, such as age-related macular degeneration or diabetic retinopathy. In this regard, the intravitreal and suprachoroidal tolerance and efficacy of Laponite has already been demonstrated. The added capacity of photothermal and photodynamic therapy could be another avenue to explore in relation to potential application of Laponite in choroidal pathology.

The eye is an organ in which the three embryological structures —endoderm, mesoderm, and ectoderm— are present. It is therefore composed of skin, connective tissue, bone tissue, blood vessels, muscle, and neural tissue [166]. Laponite has a regenerative role in mesenchymal (keratinocytes, fibroblasts, chondrocytes, osteoblasts) and vascular tissues, which is useful for the repair of damaged or lost tissue. As Laponite improves stimulus responsiveness, increases cell adhesion and differentiation, and improves scaffolds' mechanical properties, it could be applicable in lid and orbital surgery with tissue defects. Autografts or donor transplants are still the gold standards for replacing lost functional tissue. However, the biomaterial Laponite could facilitate the performance of minimally invasive procedures by simplifying injection and/or placement of scaffolds cultured with the patient's cells versus the complex reconstructive surgery that is currently performed [167]. Moreover, facial and lid surgery can produce bleeding that is sometimes difficult to stem. In this sense, Laponite could also facilitate the procedure by reducing bleeding time. However, no reviewed articles have evaluated Laponite in periocular and/or orbital tissue; most have focused on articular orthosis.

Regarding neural tissue, only four publications referring to Laponite were found. One tested Laponite on cultured medulloblastoma cells, another on mouse brain tissue, and the other two on rabbit and rat retinas. The studies carried out with Laponite in the eye demonstrated the absence of toxicity and even animal neuroretinal protection after suprachoroidal and intravitreal administration. Thus, Laponite would be a possible biomaterial to consider for retinal tissue bioprinting in future studies.

Laponite is also useful for imaging, cell tracking, or directing. These characteristics are potentially also used in ophthalmology. The Laponite–brimonidine intravitreal formulation was visualized and monitored using vitreous OCT imaging and even correlated with intraocular drug levels. In addition, Laponite can be functionalized with different markers or molecules to target and internalize the target cell and exert its beneficial and/or harmful effect (in the case of cancer). This theranostic advantage that Laponite offers could change the clinical practice and prognosis of ocular tumors or pathologies that are difficult or impossible to access in the orbit or optic nerve such as gliomas [168], which represent a high risk of iatrogenic blindness.

Finally, not only does Laponite meet the requirements stated by Ferrari et al. for nanotechnology for therapy [169,170], but also does so for ophthalmic purposes, as this review has shown: (1) it enables administration of lipophilic drugs, such as brimonidine, a common hypopressure and neuroprotective antiglaucoma drug; (2) it provides sustained targeted delivery of therapeutic agents to target cells or tissues; (3) it overcomes the epithelial and endothelial barriers due to drug transcytosis and its easy injectability; (4) it delivers large therapeutic agents (macromolecular structures such as growth factors, i.e., ILGF) to the intracellular sites of action; (5) it enables co-delivery of two or more therapeutic agents to produce synergistic action, obtained with anti-inflammatory or chemotherapy drugs but potentially used in other multifactorial pathologies such as neurodegenerative diseases; (6) it has a high circulation time compared to free drugs, thanks to drug retention with sustained delivery, which decreases cytotoxicity; (7) it improves the pharmacokinetic profile; (8) it enables visualization of therapeutic agent delivery sites by combining therapeutic agents with imaging techniques such as OCT or MRI; and (9) it allows in vivo real-time OCT monitoring of therapeutic efficacy.

Limitations on use of Laponite and future studies: The review conducted shows numerous advantages and potential applications of Laponite in ophthalmology. However, it also found a few, partially reversible limitations on use of Laponite in biomedicine which are discussed below. Regarding drug delivery, the electrostatic interactions between the nanosheets limit dispersion in aqueous media, although the addition of other compounds reduced such electrostatic interactions and accelerated the dispersion of the films [171]. There could also be potential toxicity at high concentrations. Laponite's bactericidal action is due to bacteria aggregation (limited to Gram-negative bacteria as Laponite did not cause flocculation of Gram-positive *Bacillus subtilis* bacteria nor did it bind to lipoteichoic acid from bacterial envelopes) [75]. Finally, regarding tissue engineering and scaffolding, although Laponite helps, the limited printability of the soft materials remains a challenge [172]. As regards intraocular administration, although no explicit references to limitations were found, intravitreal injection was reported to produce a transient IOP increase at around 3 days, similar to other hydrogels [49]. Although its luminescent property could produce light scattering and alter visual quality, no in vivo studies have evaluated this [173]. Finally, removal of the material in the case of allergy or intolerance would possibly require a challenging surgical intervention. Future studies should therefore analyze these issues. Furthermore, this review focuses solely on Laponite clay for ophthalmic application; comparing other types of clays with potential application in ophthalmology would also be beneficial.

## 5 Conclusion

In conclusion, this review presents the few studies carried out on application of the biomaterial Laponite in the eye and/or ocular tissue to date. However, there is extensive scientific evidence suggesting that Laponite can be used in all ocular structures and tissues, from the skin and ocular appendages to the retina and orbit. Its advantages include —in the case of ophthalmology— biocompatibility, optical transparency, nanosize thickness, and thixotropy facilitating easy injection, in addition to its capacity to retain all types of molecules, even in co-loading, and its ability to release them progressively to treat the target cell after administration in the form of topical gel or skin, intravitreal, or suprachoroidal injection, or as scaffolds. It also possesses intrinsic bactericidal and regenerative characteristics. Laponite's clinical transformation in terms of drug delivery seems more feasible, straightforward, and closer. Scaffolding, in contrast, and especially for neural tissue, seems distant, as the complex connections between retinal cell types remain a challenge. Laponite is therefore a biomaterial that merits further study in medical, surgical, and regenerative applications in future ophthalmological research.

## Patents

E.G.M., J.M.F., J.A.M., and L.E.P. are inventors on a pending European patent application (No. 20 382 021.2) related to this technology. The terms of this arrangement are being managed by the Aragon Health Research Institute (IIS Aragon), Zaragoza University and the Spanish Science Research Council (CSIC) in accordance with its conflict-of-interest policies.

## Funding

This study was supported by Grants M17/00213, JR22/00057, PI17/01726, PI17/01946, and PI20/00437 (10.13039/501100004587Carlos III Health Institute), and by MAT2017-83858-C2-1, MAT2017-83858-C2-2, PID2020-113281 R B-C2-1, and PID2020-113281 R B-C2-2 funded by (*MCIN/*10.13039/501100011033*AEI**/10.13039/501 100 011 033*).

## TOC

This is the first review to present the biomedical applications of Laponite for ophthalmology from a medical, surgical and regenerative perspective. Very few studies have been conducted in the eye, but both the findings and new treatments resulting from previous research seem potentially applicable to ophthalmology in the future.

## CRediT authorship contribution statement

**Maria J. Rodrigo:** Conceptualization, Funding acquisition, Supervision, Writing - original draft, Writing - review & editing. **Maria J. Cardiel:** Investigation, Methodology, Software, Validation, Writing - review & editing. **Jose M. Fraile:** Conceptualization, Funding acquisition, Investigation, Methodology, Writing - original draft, Writing - review & editing. **Jose A. Mayoral:** Conceptualization, Supervision, Validation, Writing - review & editing. **Luis E. Pablo:** Conceptualization, Funding acquisition, Project administration, Supervision, Writing - review & editing. **Elena Garcia-Martin:** Conceptualization, Formal analysis, Supervision, Writing - original draft, Writing - review & editing.

## Declaration of competing interest

The authors declare the following financial interests/personal relationships which may be considered as potential competing interests:Elena Garcia-Martin reports financial support was provided by 10.13039/501100004587Carlos III Health Institute. Maria J Rodrigo reports financial support was provided by 10.13039/501100004587Carlos III Health Institute. Luis E Pablo reports financial support was provided by Spain 10.13039/501100004837Ministry of Science and Innovation. Maria J Rodrigo reports was provided by Spain Ministry of Science and Innovation. Maria J Cardiel reports financial support was provided by Spain 10.13039/501100004837Ministry of Science and Innovation. Elena Garcia-Martin has patent #No. 20 382 021.2 pending to Pending European patent application (No. 20 382 021.2) related to this technology. If there are other authors, they declare that they have no known competing financial interests or personal relationships that could have appeared to influence the work reported in this paper.

## Data Availability

Data will be made available on request.
