# Supplementary material for: Laponite for biomedical applications: An ophthalmological perspective
Source: Mater Today Bio. 2023 Dec 28;24:100935. doi: 10.1016/j.mtbio.2023.100935 (PMC10794930; doi:10.1016/j.mtbio.2023.100935)
Supplement: Multimedia component 2 [file mmc2.docx]

Supplementary Table 1. Summary of studies of potential Laponite application in the eye from the last 10 years (2013–2023).

| **PUBLICATION** | **STUDY** | **MOLECULES** | **TEST ON** | **OUTCOMES** |
| --- | --- | --- | --- | --- |
| **DRUG RETENTION AND DELIVERY** | | | | |
| Review | *In vitro & In vivo* | - | - | Clay nanoparticles as pharmaceutical carriers in drug delivery systems. [57] |
| Review | *In vitro & In vivo* | - | - | 3G drug delivery nanoparticles have to overcome formulation and biological barriers. [59] |
| Review | *In vitro & In vivo* | - | - | 2D nanomaterials explored for biomedical applications include [nanoclays](https://www.sciencedirect.com/topics/engineering/nanoclays), among many others. Most of these nanomaterials are biocompatible and degrade into nontoxic products, which is advantageous for therapeutic delivery systems. [61] |
| Article | *In vitro* | GM-CSF and IL-2 |  | Injectable nanocomposite hydrogel. By modifying the Laponite content within the hydrogel, the kinetics of several proteins’ release could be precisely tuned. [60] |
| Article | *In vitro* | - | - | The addition of Laponite as a second crosslinker together with a divinyl monomer strongly enhanced the materials’ properties. The hydrogels described here may find applications for water purification and in the biomedical field as drug delivery systems or wound dressings. [62] |
| Article | *In vitro* | Dexamethasone | MG63 cells | Laponite nanoplates as a platform for the efficient release of anionic dexamethasone, which was encapsulated into the interlayer space. Release of dexamethasone was pH-dependent, and at a faster rate at acidic pH than at a physiological pH. It also showed cytocompatibility in MG63 cells. [64] |
| Article | *In vitro* | - | - | Investigates the mechanism of degradation of Laponite. Increase in concentration of Laponite as well as that of salt, having a stabilizing effect against degradation. [65] |
| Article | *In vitro* | Bovine serum albumin, human serum albumin and β-lactoglobulin | - | Morphology-dependent interaction of Laponite with three globular plasma proteins. Acidic residues of these proteins were adsorbed onto the platelet surfaces. [66] |
| Article | *In vitro* | Tetracycline | Simulated saliva solution | Laponite clay as a carrier for in situ delivery of tetracycline over a 72-hour period for treatment and prevention of periodontal disease. [67] |
| Article | *Ex vivo* | Ciprofloxacin | Simulation of subcutaneous pig tissue | Topical photothermal hydrogel for near-infrared-controlled drug delivery loaded with ciprofloxacin. Laponite content increased the density of crosslinking in the hydrogel, which improved its mechanical properties noticeably. It also showed excellent antibacterial effect vs. *E. coli* and *S. aureus*, and good blood compatibility. [69] |
| Article | *In vitro* | Mafenide | Skin fibroblast culture | Laponite in combination with the antimicrobial agent (mafenide). It has the capacity to absorb wound exudates and showed benefits in wound-healing processes by releasing Mg(2+) ions, and also reduced the cytotoxic effect of mafenide on fibroblast cells. [70] |
| Article | *In vitro* | Chlorhexidine | GM07492A cells (human fibroblasts) | Aminofunctionalized Laponite as a versatile hybrid material for controlled chlorhexidine digluconate release. It was effective against S. pyogenes. [71] |
| Article | *In vitro* | NO | Endothelial cell tube | Laponite was used to fabricate an NO delivery carrier through the formation of Laponite–polyamine composites that allowed local release of the NO gas at the site of application and showed cell viability. [72] |
| Article | *In vitro* | Cu | Mouse bone marrow stromal cells | Scaffold made of Laponite–copper–poly(butylene succinate) could inhibit the growth of both *E. coli* and *S. aureus*, indicating good antibacterial activity. It also significantly promoted proliferation and activity of bone mesenchymal stem cells. [73] |
| Article | *In vitro* | Ag or Cu or Zn | - | A thin film with Laponite (Ag or Cu or Zn) synthesized by the ion exchange method showed effective bactericidal properties against both E. coli and S. aureus. [74] |
| Article | *In vitro* | - | - | Laponite nanoparticles did not kill Gram-negative *E. coli* bacteria or disrupt anionic model liposomes. They did however cause bacteria flocculation, originating from the interaction of Laponite and bacterial lipopolysaccharide. [75] |
| Article | *In vitro* | Theophylline | Simulated gastric and intestinal fluid | Intercalation of theophylline in Laponite included in sodium alginate beads achieved pH-controlled drug release, which prevented the release of theophylline at low pH in simulated gastric fluid and resulted in sustained release in simulated intestinal fluid, with 40% dissolution within 120 min. [76] |
| Lipophilicity | | | | |
| Article | *In vitro* | ITH12657 | Media mimicking the gastrointestinal tract | Investigation of the use of Laponite as a vehicle for the gramine-based neuroprotectant ITH12657. Oral administration and controlled delivery of the neuroprotective drug did not exert toxicity in cell cultures and provided neuroprotection. [79] |
| Article | *In vitro* | - | - | Use of a Laponite dispersion to increase the hydrophilicity of cobalt–ferrite magnetic nanoparticles. [80] |
| **Proteins and other macromolecules** | | | | |
| Article | *In vitro* | GM-CSF and IL-2 | - | Injectable nanocomposite hydrogel. By modifying the Laponite content, the kinetics of several proteins’ (GM-CSF and IL-2) release could be precisely tuned. [60] |
| Article | *In vitro* | Extracellular vesicles | Human bone marrow stromal cells | Gelatin–methacryloyl–Laponite hydrogel to improve local retention and control delivery of epigenetically enhanced osteoblast-derived extracellular vesicles. Laponite increases compressive modulus and shear-thinning properties to enhance shape fidelity when 3D printed. It increases mineralization capacity, releasing significantly more extracellular vesicles as a novel bone repair strategy. [83] |
| Article | *In vitro & In vivo* | Extracellular vesicles | Primary articular cartilage, chondrocytes from mice and knee joint from rats | Gelatin methacrylate nanoclay hydrogel for sustained release of extracellular vesicles, which was biocompatible and exhibited excellent mechanical properties and promoted cartilage regeneration. [84] |
| Article | *In vitro & In vivo* | Insulin-like growth factor-1 mimetic protein | Rodent model of Achilles tendon injury | Composite clay hydrogels, which simultaneously achieve a spectrum of mechanical, storage, and drug loading/releasing properties to address the critical needs from translational perspectives while demonstrating biodegradation and biocompatibility. Storage and extended release of large quantities of an insulin-like growth factor-1 mimetic protein over four weeks. [85] |
| Article | *In vitro & In vivo* | VEGF | Cell culture tubulogenesis and angiogenesis assays | Injectable Laponite gel can stabilize VEGF and retain it in the active form for therapeutic delivery. Enhanced tubulogenesis in a dose-dependent manner in vitro. Administered subcutaneously in vivo, Laponite was retained at the injection site for up to 3 weeks and promoted a 4-fold increase in blood vessel formation. It provides a robust method for delivery of bioactive recombinant VEGF without the need for complex hydrogel or protein engineering. [86] |
| Article | *In vitro & In vivo* | FGF4 | Primary neuron and spinal cord of rats | Laponite/Heparin/FGF4 revealed remarkable motor functional recovery and axonal regrowth after spinal cord injury by suppressing inflammatory reaction, increasing remyelination, and reducing glial/fibrotic scars, enhancing microtubule stability and regulating mitochondrial localization. Laponite hydrogel delivery at the injured site was a promising strategy to promote axon regeneration and motor function recovery. [87] |
| Article | *In vitro & In vivo* | BMP-2 | Subcutaneous implantation in mice | Specific bisphosphonate interactions with the positively charged clay particle edge to develop self-assembling hydrogels with enhanced mechanical properties and with preserved protein-binding surface exchange capacity able to sustain the growth factor BMP-2 over 6 weeks. [88] |
| Article | *In vitro* | rhBMP2 and TGFb | Human mesenchymal stem cells | The Laponite composite shows high binding efficacy without altering protein formation, bioactivity, or delivery of active proteins (rhBMP2 and TGFb) for more than 30 days. It enhanced differentiation of human mesenchymal stem cells at a 10-fold lower concentration. Nanosilicates as a delivery vehicle could minimize the negative side effects observed because of the use of supraphysiological dosages of protein therapeutics for orthopedic regeneration. [89] |
| Article | *In vitro* | Factor-rich stem cell secretome | - | A nanocomposite hydrogel with photocrosslinkable micro-porous networks and nanoclay release of growth factor-rich stem cell secretome showed pro-angiogenic and cardioprotective potential. [90] |
| Article | *In vitro* | BMP2 | Myoblasts | Laponite localized and enhanced the activity of BMP2, which enhanced osteogenic differentiation in in vitro myoblasts at doses within the sub-microgram per ml range of concentrations sufficient to induce differentiation of responsive cell populations at levels approximately 3000-fold lower than those employed in clinical practice. [92] |
| **Anti-cancer therapy** | | | | |
| Review | *In vitro & In vivo* | - | - | Nanoclays may have a wide range of applications in oncology. Their submicron size, individual morphology, high specific surface area, enhanced adsorption ability, cation exchange capacity, and multilayered organization of 0.7–1 nm thick single sheets have attracted considerable interest as multifunctional biocompatible nanocarriers with versatile applications in cancer research, diagnosis, and therapy. [93] |
| Review | *In vitro & In vivo* | - | - | 2D nanomaterials have been widely studied, showing promising applications in cancer therapy and diagnosis, including phototherapies, magnetic therapy, drug and gene delivery, and non-invasive imaging. Physically triggered nanosystems based on graphene and two-dimensional nanomaterials. The physical triggers include light, temperature, and magnetic and electric fields. [94] |
| Review | *In vitro & In vivo* | - | - | Discusses the latest nanotechnology applications (such as bionic nanoparticles, self-assembled nanoparticles, deformable nanoparticles, photothermal-effect nanoparticles, stimuli-responsive nanoparticles, and other types) in cancer immunotherapy. [95] |
| Article | *In vitro* | Doxorubicin | Primary lung epithelial cells | Laponite–c poly(ethylene glycol)–doxorubicin formulation was transparent and maintained liquid-like homogeneity without delamination and higher drug loading efficiency. The results of the cell viability assay indicated that the LAP-cPEG/DOX formulation could effectively inhibit the proliferation of A549 lung carcinoma epithelial cells. [96] |
| Article | *In vitro & In vivo* | Cisplatin, 4-fluorouracil, and cyclophosphamide | Human breast adenocarcinoma cells (MCF-7), human cervix adenocarcinoma cells, and rats | New nanohydrogel drug-delivery platform based on Laponite. Soft, flexible, biocompatible, biodegradable, nonswellable, pH-responsive, noncytotoxic, and able to deliver antineoplastic drugs into cancer cells. Cisplatin, 4-fluorouracil, and cyclophosphamide are significantly lower than the IC50 of the free drugs. Simultaneous encapsulation of several cancer drugs yielding an efficient drug cocktail delivery system, with a positive synergistic effect against MCF-7 cells. [97] |
| Article | *In vitro* | Doxorubicin | Human epithelial carcinoma cells, KB cells | Enhanced antitumor efficacy is primarily due to the greater cellular uptake of the Laponite/doxorubicin nanodiscs. Doxorubicin is released at a quicker rate at acidic pH. [98] |
| Article | *In vitro* | Doxorubicin | MCF-7 cells | Simple method to modulate drug release at different pH values. Polyelectrolyte multilayers improved the sustained release properties of Laponite and allowed fine-tuning of the extension of drug release at neutral and acidic pH values. Doxorubicin/Laponite nanoparticles can be effectively internalized by cells leading to Doxorubicin accumulation in the cell nucleus. [99] |
| Article | *In vitro* | Doxorubicin | CAL-72 cells | Laponite/Doxorubicin/Alginate nanohybrids exhibit biocompatibility, high loading capacity, stimulus-responsive release of cationic chemotherapeutic drugs, and remarkably higher cytotoxicity by effective internalization in CAL-72 cancer cells (an osteosarcoma cell line). [100] |
| Article | *In vitro* | - | Macrophage line cells | New Raman imaging methodology to track the uptake and internalization of Laponite nanoparticles into J774 macrophage line cells is presented in this paper through its unique vibrational fingerprint without labelling or adding dyes, and taking advantage of the fact that Laponite and biological molecule bands can be clearly differentiated. [101] |
| Article | *In vitro* | Simvastatin | Melanoma | New Laponite gel containing the drug simvastatin targeting topical treatment of melanoma. The amount of drug permeated through the human skin (epidermis+dermis) after 24 h was lower than 0.01% of the total initial amount. [102] |
| Article | *In vitro* | 5-fluorouracil | - | Magnetite–Laponite hydrogel composition and structure, thermoresponsive properties, hydrogel sorption, and release of 5-fluorouracil. All hydrogel nanocomposites have a distinct volume phase transition from a swollen state to a collapsed state upon heating within the physiologically acceptable temperature range of 33–36 °C. [103] |
| Article | *In vitro* | O2 | Primary dermal fibroblasts and human Colo 818 (malignant melanoma) cells | New oxygen-generating organic peroxide-based injectable 3D biomaterial, prepared by using benzoyl peroxide and Laponite incorporated into an alginate hydrogel, shows sustained release of O_2_ over a period of 14 days and reduces hypoxia-induced cell death, decreases proliferation of malignant cells, and increases the viability of healthy fibroblast cells. [105] |
| Article | *In vitro* | Doxorubicin and methotrexate | HepG2 (a human liver cell line) | Loading of different anticancer drugs (doxorubicin and methotrexate) via step-by-step assembly, where variation of each drug amount can be used for adjustment of the sizes of the resulting nanocomplexes. Dual-drug loaded nanosystems allow sequential release. Drug release can be accelerated under acidic and heating treatment, becoming synergistic. It presented 26 times’ higher antitumor cytotoxicity than the corresponding single-drug systems. [106] |
| Article | *In vitro* | Doxorubicin | HeLa cells with CD44 receptors overexpressed | Laponite/hyaluronic acid/doxorubicin showed high drug-loading efficiency, pH-sensitive drug-release properties, and CD44 targetability-mediated endocytosis. It might be an efficient nanoplatform for cancer chemotherapy. [107] |
| Article | *In vitro* | ROS | Human breast cancer MDA-MB-231 cells | Novel photothermal and photodynamic therapeutic nanoplatform with reactive oxygen species generating the ability to treat cancer cells overexpressing integrin αvβ3 through the coating of polydopamine on indocyanine green-loaded Laponite and then further conjugating polyethylene glycol–arginine–glycine–aspartic acid. Indocyanine green-loaded Laponite exhibited much better photothermal stability than free. [109] |
| **BLEEDING** | | | | |
| Article | *In vitro & In vivo* | - | Pig red blood cells | Laponite possesses excellent surface hydrophilicity and serum absorption capacity, good cytocompatibility, and hemocompatibility in hemolytic assay of pig red blood cells. All materials formed under different sintering temperatures have hemolysis percentages lower than 5% while Laponite compacted without sintering shows a slight hemolysis effect (hemolysis percentage of 8.3 ± 0.2%). Sintering gives the Laponite improved hemocompatibility and degradation products. Relatively high concentration may generate a certain degree of hemolysis. [39] |
| Article | *Ex vivo* | - | Rabbit red blood cell and subcutaneous pig tissue | A topical photothermal antibacterial hydrogel showed a blood hemolysis ratio of less than 5%, signifying good blood compatibility. [69] |
| Article | *In vitro & In vivo* | - | Red blood cells and subcutaneous tissue in bleeding liver model in rats | Shear-thinning nanocomposite hydrogels composed of Laponite and gelatin as injectable hemostatic agents. Injectability, rapid mechanical recovery, physiological stability, and the ability to promote coagulation result in a hemostat for treating incompressible wounds. Nanocomposites with higher nanoplatelet loadings clearly highlight the presence of a clot earlier. This was attributed to the strong negative charge of Laponite, which can facilitate concentration of clotting factors near the nanocomposite surface. [111] |
| Article | *In vitro* | - | Human umbilical cord endothelial cells, platelets, and red blood cells | Injectable nanoengineered hemostats. Nanosilicates mechanically reinforce the hydrogels, provide enhanced physiological stability, increase protein adsorption that results in enhanced cell adhesion and spreading, increase platelet binding, and accelerate clotting time two-fold. They significantly suppress the release of trapped vascular endothelial growth factors and facilitate tissue regeneration and wound healing. [113] |
| Article | *In vitro* | - | Rabbit blood | Novel hemocompatible nanocomposite hydrogel by in situ polymerization of acrylamide in a mixed suspension containing Laponite and gelatin. It exhibited good thermal stability and mechanical properties, resisted nonspecific protein adsorption, improved the degree of hemolysis, and eventually prolonged the clotting time. The addition of gelatin improved the pH-responsive properties, enhanced the antithrombogenicity, and decreased the extent of hemolysis. [114] |
| **TISSUE ENGINEERING: REGENERATIVE MEDICINE AND SCAFFOLDS** | | | | |
| Review | *In vitro & In vivo* | - | - | Overview of how to design efficient delivery of therapeutics by leveraging the properties and specific interactions of various Laponite–polymer composites and drug moieties. [15] |
| Review | *In vitro & In vivo* | - | - | Summarizes the existing inorganic biomaterials-based bioinks (referred to as “inorganic-bioinks”) for 3D-bioprinting regenerative scaffolds. It especially highlights that the incorporation of inorganic biomaterials improves printability, mechanical strength, and bioactivity in different tissue regeneration applications. [119] |
| Review | *In vitro & In vivo* | - | - | Current research into tissue engineering focuses on the development of compatible methods (printers) and materials (bioinks) capable of producing biomimetic scaffolds. An overview of current 3D printing techniques used in tissue engineering is provided with an emphasis on the printing mechanism and the resultant scaffold characteristics. [122] |
| Review | *In vitro & In vivo* | - | - | Provides the specific considerations regarding the important properties of a potential bioink and the generated 3D construct, including rheological, interfacial, structural, biological, and degradation properties, which are crucial for printing of complex and functional 3D structures. Interfacial bonding seems to be a key consideration in successfully obtaining a 3D structure. [123] |
| Review | *In vitro & In vivo* | - | - | The field of stiffness gradient hydrogel fabrication methods, mechanical property tests, and applications. Different cell behaviors depending on model stiffness gradient hydrogel system. [124] |
| Review | *In vitro & In vivo* | - | - | How to develop printable materials and make unprintable materials printable, to choose suitable methods, improve printing resolution, and to directly construct functional structures/systems with 3D printing. [127] |
| Review | *In vitro* | - | - | Focus on recent promising advances in the development of 3D-printed hydrogels containing polymeric nanofibers that can improve cell-material interaction in biomedical applications. [131] |
| Review | *In vitro* | - | - | State-of-the-art of a particular type of heterogeneous bioinks, which are composed of polymeric hydrogels incorporating inorganic bioactive fillers and include isotropic and anisotropic silicates like bioactive glasses and nanoclays or calcium-phosphates like hydroxyapatite, which provide in situ crosslinking effects and add extra functionality to the matrix. For example, mineralization-capable bioactive fillers are a promising addition to hydrogels to print stable 3D constructs for tissue regeneration. [132] |
| Review | *In vitro & In vivo* | - | - | Potential role of clays‘ key physicochemical properties in influencing such interactions for regenerative medicine. Polymeric nanocomposites for self-assembly and enhanced mechanical properties as well as for their potential as drug delivery modifiers, bioactive additives able to enhance cellular functions including adhesion, proliferation, and differentiation, most notably for osteogenesis. High surface reactivity of clays and their wide range of possible interactions with polymers, proteins, and minerals makes this an exciting area in biomaterial design. [155] |
| Article | *In vitro* | - | Keratinocyte cells | Bacterial cellulose Laponite composite membranes, with 3D hierarchical organization, cell viability, and transparency for biomedical, pharmaceutical, and cosmetics uses. [37] |
| Article | *In vitro* | - | Human mesenchymal stem cells | Novel silicate nanoplatelets that induce osteogenic differentiation of human mesenchymal stem cells in the absence of any osteoinductive factor. [41] |
| Article | *In vitro* | - | - | Laponite/alginate mixtures for 3D printing showed pronounced shear-thinning behavior for filament formation with Laponite concentrations of at least 5 wt% with instant structure recovery and therefore compatible with open-source 3D printers. [115] |
| Article | *In vitro* | - | - | A 4D printing hydrogel was fabricated by in situ polymerizing acrylamide in the agarose matrix containing Laponite. Laponite played an important role in achieving shear-thinning behavior to allow easy extrusion and excellent shape stability after printing. [116] |
| Article | *In vitro & In vivo* | - | Pre-osteoblast murine bone marrow stromal cells and subcutaneous tissue in rats | A shear-thinning and printable hydrogel based on Laponite and glycosaminoglycan nanoparticles for bioprinting which protected encapsulated cells and showed fast recovery over multiple strain cycles, biocompatibility, and biodegradability. [117] |
| Article | *In vitro* | - | L929 fibroblasts | Technological development of a hydrogel-based Laponite and alginate ink and evaluation of its printing capacity stability over time and absence of cytotoxicity. Provides new printability parameters such as the Printing Accuracy Escalation Index. [118] |
| Article | *In vitro* | - | - | Novel self-supporting direct hydrogel printing approach to extrude complex 3D hydrogel composite structures in air without the help of a support bath. Laponite can be easily extruded through a nozzle as a liquid and self-supported after extrusion as a solid. Increasing the Young's modulus therefore improves the mechanical and biological properties of hydrogel composites. [121] |
| Article | *In vitro* | - | HeLa and NIH-3T3 cells | Bioactive nanocomposite hydrogels based on agarose and Laponite were developed as strong shear-thinning bioinks for extruded 3D bioprinting applications. The interaction between Laponite nanosilicates and agarose chains led to higher elastic moduli (*G′*) of nanocomposite hydrogels and the gelation temperature produced 3D printed structures with high shape fidelity and structural integrity. Nanocomposite bioinks showed significant improvement in metabolic activity of encapsulated cells, resulting in full spreading and elongation of fibroblasts, and the extent of change in cell morphology was found to be directly correlated with nanosilicate concentration. [128] |
| Article | *In vitro* | - | Bovine serum albumin and vascular endothelial growth factor and  human mesenchymal stem cells | Laponite–alginate–methylcellulose achieving scaffolds with high printing fidelity. Laponite improved printability, increased shape fidelity, and was beneficial for controlled release of biologically active agents such as growth factors. Cell viability was maintained over 21 days. [129] |
| Article | *In vitro & Ex vivo* | VEGF | Human bone marrow stromal cell and chick chorioallantoic membrane model | Novel light-curable nanocomposite bioink for 3D printing for skeletal regeneration with potential application in hard and soft tissue repair. Laponite with a gelatin methacryloyl bioink enhanced shape fidelity retention and interconnected porosity within extrusion-bioprinted fibers. Support of cell growth was evidenced as a significant increase in cell number over 21 days. Scaffolds supported osteogenic differentiation evidenced by mineralized nodule formation, including in the absence of the osteogenic drug dexamethasone. Growth factor retention and delivery of loaded VEGF generated higher vessel penetration evidenced by ex vivo vasculogenesis. [130] |
| Article | *In vitro & In vivo* | Collagen, heparin, and human dermal fibroblast cells | Mouse myoblast cells within a mouse skeletal muscle injury model | Nanocomposite injectable gels possess unique abilities to self-replenish the regenerative extracellular microenvironment within the gels in the body, demonstrating potential utility for in vivo tissue engineering. They strongly adsorb molecules, including collagen and heparin, within gels and retain them due to the ability of the Laponite in synchronization with the degradation of PLGA-PEG-PLGA and the subsequent release of the degradation products. High cell viability and proliferation for at least a week. Cells encapsulated within the nanocomposite gels exhibited significantly higher survival, proliferation, and three-dimensional organization. Enhanced tissue regeneration and functional recovery were reported. [133] |
| Article | *In vitro* | - | Human mesenchymal stem cells | Transcriptomic insight on the role of surface-mediated cellular signaling triggered by nanomaterials and development of nanomaterials-based therapeutics for regenerative medicine. A widespread alteration of genes was observed due to nanosilicate exposure as more than 4,000 genes were differentially expressed, revealing clathrin-mediated endocytosis, activated stress-responsive pathways such as mitogen-activated protein kinase, and human mesenchymal stem cell differentiation toward osteogenic and chondrogenic lineages. [134] |
| Article | *In vitro* | - | Fibroblast cells | Nanocomposite scaffolds based on chitosan–Laponite were produced for potential application in skin regeneration and wound dressings. The porous architecture, besides the increase in the clay content, leads to an increase in porosity, an improvement in mechanical strength, and a decrease in swelling capacity. Cell adhesion and viability were demonstrated. Scaffolds were not cytotoxic and the fibroblast cells readily attached to the surface of the scaffolds. [135] |
| Article | *In vitro* | Type I collagen |  | Laponite was surface-functionalized via covalent bonds with tetrakis(hydroxymethyl) phosphonium sulfate to reinforce its incorporation into the type I collagen matrix. It stabilizes the intrinsic triple-helical formation of the collagen, conferring improved thermal stability and enhanced mechanical properties. [136] |
| Article | *In vitro & In vivo* | - | Osteochondral tissue and rat osteochondral defect model | Synthesis of a bilayered Laponite–alginate–polyacrylamide composite hydrogel and investigation of immunocompatibility. The cartilage layer was formed by alginate polyacrylamide with and without the addition of TGF-β3, and the bone layer was formed by Laponite. It activated macrophages towards the M2 phenotype and stimulated the expression of anti-inflammatory factors. A bilayer structure exhibited better cell viability as well as repair through the Laponite content. Surface regularity and hyaline-like tissue formation, along with a synchronized degradation profile of the hydrogel with tissue healing at the end of 12 weeks. [137] |
| Article | *In vitro* | - | - | Laponite stability versus dissolution in its aqueous dispersions is investigated as a function of initial water pH. Dissolution is observed even though the dispersion pH is above 10 for low concentrations of Laponite. Conversely, for dispersions with high concentrations of Laponite no dissolution was observed. [142] |
| Article | *In vitro & In vivo* | Bone mesenchymal stem cells | Rat bone defects | The effect of 3D-bioprinted Laponite hydrogel protected encapsulated bone mesenchymal stem cells from shear stresses during bioprinting, promoted cell growth and cell spreading, and favored optimal osteogenesis potential by activating the PI3K/AKT signaling pathway and through upregulated expression of osteogenic related proteins. The 3D-bioprinted nano-Laponite hydrogel construct exhibited superior bone regeneration ability in rat bone defects. [148] |
| Article | *In vitro* | - | Adipose-derived cell-subpopulation | Interactions between adipose-derived cell-subpopulation and Laponite. Laponite below 100 μg/mL showed high cytocompatibility and fast internalization via a clathrin-mediated pathway and triggered overexpression of osteogenic-related markers and increased alkaline phosphatase activity and deposition of a predominantly collagen-type I matrix. Osteogenic differentiation potential was enhanced by the addition of Laponite in a dose-dependent manner. [150] |
| Article | *In vitro* | - | Human bone marrow stromal cells | To explore the role of lithium in the modulation of Laponite bioactivity. Nanoparticles are biocompatible and promote early osteogenic activity in human bone marrow stromal cells. Up to a concentration of 100 µg mL−1 they robustly enhance certain bone-related phenotypic changes without loss of viability. Results suggest that other properties of Laponite nanoparticles, and not their lithium content or the involvement of the canonical Wnt pathway, are responsible for their bioactivity. [152] |
| Article | *In vitro* | - | Human skeletal progenitor cells and human bone marrow stromal cells | Laponite and extracellular matrix multilayer nanofilms were successfully generated through electrostatic and protein–clay interactions. Application of a NaCl solution resulted in the generation of stable, multi-stacked Laponite layers which displayed enhanced mechanical properties able to sustain human skeletal progenitor cell growth and enhanced osteogenic differentiation potential as a consequence of the synergistic effects, which suggest a potential application in hard tissue engineering. [153] |
| Article | *In vitro* | - | Human umbilical cord Wharton's jelly-derived mesenchymal stem cells | A hydrogel coating consisting of gelatin methacryloyl-methacrylamide-modified ε-poly-l-lysine and Laponite on PEEK through UV-initiated crosslinking improved hydrophilicity, degraded slowly for 8 weeks, enhanced viability and adhesion of cultured human umbilical cord Wharton's jelly-derived mesenchymal stem cells, and improved induction of osteogenic differentiation. [154] |
| Article | *In vitro* | - | Preosteoblasts | Elastomeric fiber-shaped cellular constructs made from poly(ethylene glycol) diacrylate, Laponite, and gelatin methacrylate via ionic and covalent crosslinking with tunable mechanical and degradation properties, cell spreading, survival, and proliferation. Nanocomposite microfibers with tunable cell adhesion characteristics designed to engineer complex tissue structures. Applications for such a system ranges from nerve tissue regeneration to blood vessel formation. [157] |
| Article | *In vitro* | - | SH-SY5Y neuroblastoma cell line | Neural cytotoxicity of a novel zwitterionic sulfobetaine hydrogel system with Laponite crosslinker. The material exhibits both thixotropic and shear-thinning behaviors, which makes it suitable for extrusion. However, elastic modulus optimization is required. It showed cell viability and growth on the hydrogel surface with extended neurites. [159] |
